# VNIR/SWIR Multispectral Polarimetric Imager for Polymer Discrimination and Identification

**DOI:** 10.3390/s26072040

**Published:** 2026-03-25

**Authors:** Ramon Prats Consola, Adriano Camps

**Affiliations:** 1 Universitat Politècnica de Catalunya—BarcelonaTech, CommSensLab-UPC, Department of Signal Theory and Communications, 08034 Barcelona, Spain; adriano.jose.camps@upc.edu; 2 IEEC—Institut d’Estudis Espacials de Catalunya, 08034 Barcelona, Spain; 3 College of Engineering, United Arab Emirates University, Al Ain P.O. Box 15551, United Arab Emirates

**Keywords:** multispectral imaging, polarimetry, polymer discrimination, spectral unmixing, stokes parameters

## Abstract

This work presents a portable polarimetric multispectral imaging (PMSI) system operating in the visible to shortwave infrared range (VNIR–SWIR: 400–1700 nm) and its application to target detection, discrimination from aquatic backgrounds, and polymer identification. The instrument integrates two synchronized cameras with motorized bandpass filters and piezoelectric polarization control, enabling the acquisition of 48 wavelength–polarization measurements per capture. This configuration allows the extraction of both intensity-based and polarimetric features, including the degree of linear polarization (DoLP). A complete radiometric and polarimetric calibration framework is implemented, encompassing system response characterization, polarization-dependent gain correction, and reflectance normalization under variable illumination. Experiments conducted on a representative set of 16 polymer materials show that polarimetric information consistently improves class separability compared to intensity-only features, with a mean gain of 6.9 (95% CI: 6.35–8.47). Although the correlation between intensity- and DoLP-based separability is moderate (r = 0.44), the results indicate complementary identification capability. Material recoverability was further evaluated using spectral unmixing techniques (VCA, N-FINDR, and PPI), with VCA offering the best accuracy–complexity trade-off on the calibrated Stokes reflectance dataset. Despite these gains, identification among chemically similar polyethylene variants remains challenging due to limited spectral and polarimetric contrast. An underwater detectability study under natural illumination reveals strong wavelength-dependent constraints: SWIR penetration is limited to 4 cm, whereas VNIR bands (430–550 nm) preserve detectability up to 20 cm, with DoLP enhancing edge visibility. These results motivate future validation in more complex aquatic conditions and with increased spectral dimensionality.

## 1. Introduction

Plastic pollution in rivers, coastal areas, and oceans is one of the major environmental problems of the 21st century. Large plastic objects and microplastics accumulate in aquatic environments, harm biodiversity, disturb ecosystems, and may also affect human health through the food chain [1,2,3,4]. Detecting plastic debris in natural waters is difficult, especially when the objects are fragmented, partly submerged, covered by biofouling, or mixed with organic matter. In these cases, conventional RGB images and intensity-based remote sensing methods often do not provide enough contrast to separate plastics reliably [5].

**Figure 1:**
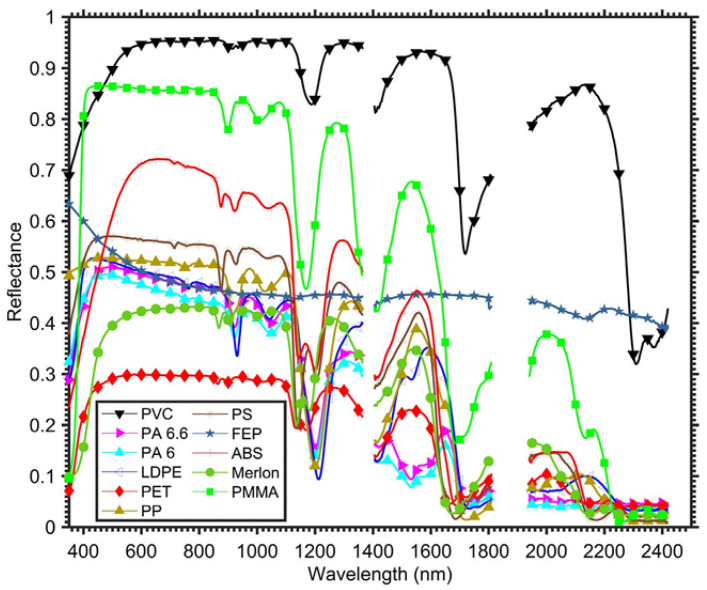
Spectral reflectance signatures of representative plastic polymers in the VNIR–SWIR range (400–2400 nm). Their characteristic absorption features help select wavelengths for polymer discrimination from Garaba et al. [6].

Spectral imaging (SI) offers a more robust way to distinguish materials because it uses their wavelength-dependent reflectance. Spectral imaging samples the electromagnetic spectrum in several bands and has shown strong potential for identifying polymers in laboratory and airborne experiments [6,7,8,9,10]. Plastics have characteristic absorption features in the visible and near-infrared (VNIR) and shortwave infrared (SWIR) ranges, mainly linked to vibrational overtones of molecular bonds. Figure 1 shows representative reflectance signatures of common plastics from 400 to 2400 nm [6]. These spectral features are the basis for polymer discrimination.

Multispectral imaging (MSI) is a practical solution because it uses a limited number of carefully chosen spectral bands. This allows systems to be smaller, faster, and easier to use in real environments. Hyperspectral imaging (HSI), in contrast, samples hundreds of contiguous bands and provides much higher spectral resolution [11], but it usually requires more complex hardware, generates larger data volumes, and is less suitable for portable field systems. Several studies have shown that a reduced set of well-selected multispectral bands can still capture the main spectral features needed for polymer identification. For example, Kikaki et al. [12] used multispectral time-series data from Sentinel-2 and Landsat-8 to monitor floating debris, while Balsi et al. [13] showed that selected SWIR bands can capture key polymer absorption features reported previously [6]. These results show that reduced-band systems can be useful in real applications.

A major limitation in aquatic environments is water absorption. The absorption coefficient of water increases strongly above about 1000 nm, which causes rapid attenuation of SWIR radiation for submerged objects [14,15,16]. As a result, SWIR bands are very useful for identifying dry plastics, but VNIR bands are more effective for partially submerged targets because they penetrate water better. Therefore, the choice of wavelengths must balance material discrimination and transmission through water.

**Figure 2:**
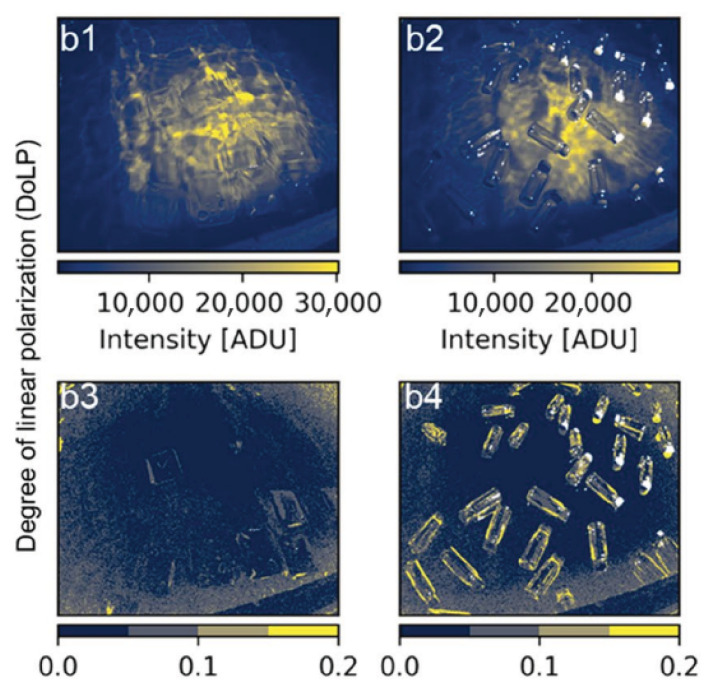
Underwater plastic discrimination using intensity and degree of linear polarization (DoLP). Polarimetric processing improves detectability compared with intensity-only images from de Fockert et al. [17].

Polarization adds another useful source of information. Plastic surfaces often produce partially polarized reflections because of Fresnel effects and surface microstructure, especially under strong glint conditions. De Fockert et al. [17] showed that the degree of linear polarization (DoLP) can make floating plastic litter more visible than intensity images alone. As shown in Figure 2, polarimetric processing can improve the contrast between plastic targets and water when spectral or intensity differences are weak.

At larger scales, satellite multispectral missions such as Sentinel-2 (MSI) and Sentinel-3 (OLCI) provide global coverage and have been used to detect floating marine litter using spectral indices and spectral unmixing methods [2,18,19]. However, their limited spectral resolution, coarse spatial resolution (10–300 m), and lack of polarization information limit their ability to identify specific polymers or detect small objects. On the other hand, laboratory studies can achieve high identification accuracy, but they are usually not designed for portable field use [7,8,20]. Some recent studies have started to combine spectral and polarimetric sensing [21,22], but compact systems that jointly optimize multispectral band selection and polarization measurements in both VNIR and SWIR are still uncommon.

In summary, previous work has shown that spectral signatures are effective for identifying polymers in controlled conditions, multispectral systems are useful for large-scale monitoring, and polarization can improve target detection in difficult viewing conditions. However, there is still a gap between laboratory characterization and compact systems that can work outdoors and capture both spectral and polarimetric information.

**Figure 3:**
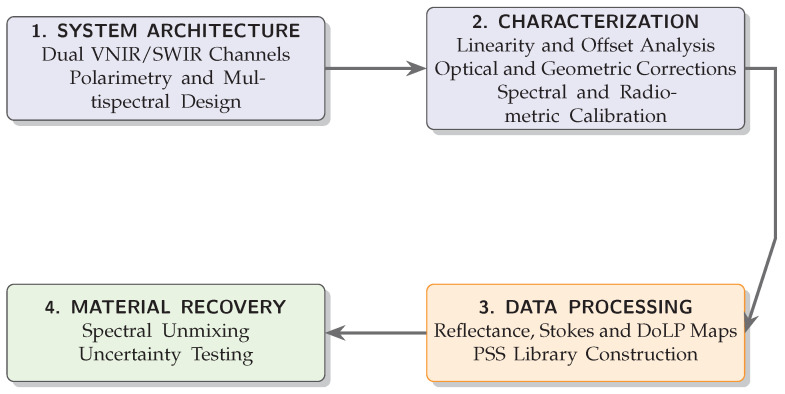
Simplified workflow of the proposed methodology, from system design to experimental validation.

This work addresses that gap by developing a synchronized dual-channel VNIR–SWIR polarimetric multispectral imaging system that acquires spectral–polarimetric data cubes under real environmental conditions. Instead of using dense hyperspectral sampling, the proposed system relies on a limited set of carefully selected spectral bands together with polarization-resolved measurements. In this way, it preserves the most relevant material information while remaining suitable for field deployment. The system is therefore intended as an intermediate step between laboratory-based plastic identification and large-scale environmental monitoring. Figure 3 provides an overview of the proposed methodology and the structure of the paper.

The main contributions of this work are:

- The design of a compact dual-channel VNIR/SWIR polarimetric multispectral imager that acquires 48 wavelength–polarization images per data cube under outdoor conditions.
- An end-to-end calibration framework, including radiometric, spectral, and polarimetric calibration, that produces absolute Stokes reflectance maps and a polymer spectral–polarimetric signature (PSS) library.
- A quantitative demonstration that DoLP features improve the separability between materials compared with intensity-only reflectance.
- Experimental validation under both dry and submerged conditions, showing the wavelength-dependent limitations caused by water absorption.

## 2. System Architecture

**Figure 4:**
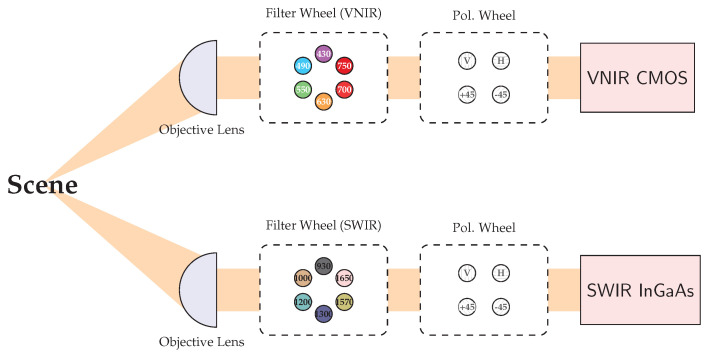
Optical layout of the dual-channel polarimetric multispectral imager (PMSI). The steup features synchronised VNIR (CMOS) and SWIR (InGaAs) cameras sharing a common scence.

**Figure 5:**
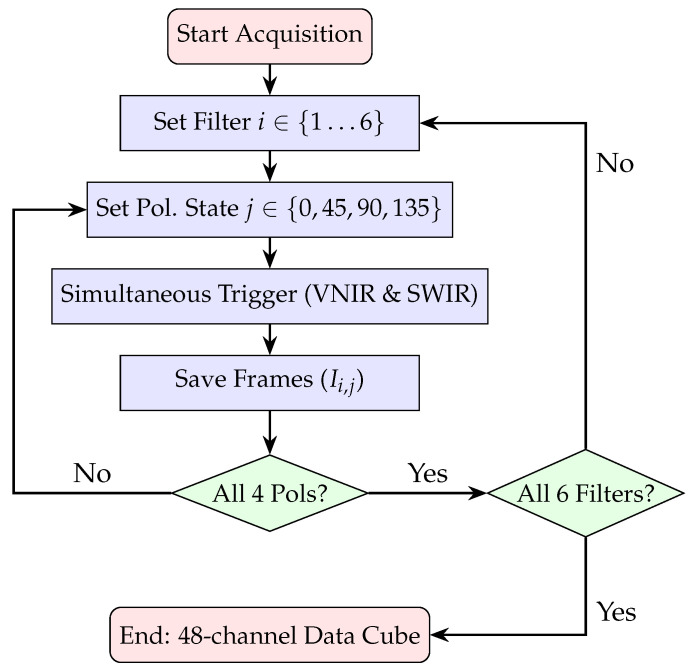
Software acquisition workflow and hardware control logic. The process implements nested loops to automate the capture of 48 unique image states.

**Figure 6:**
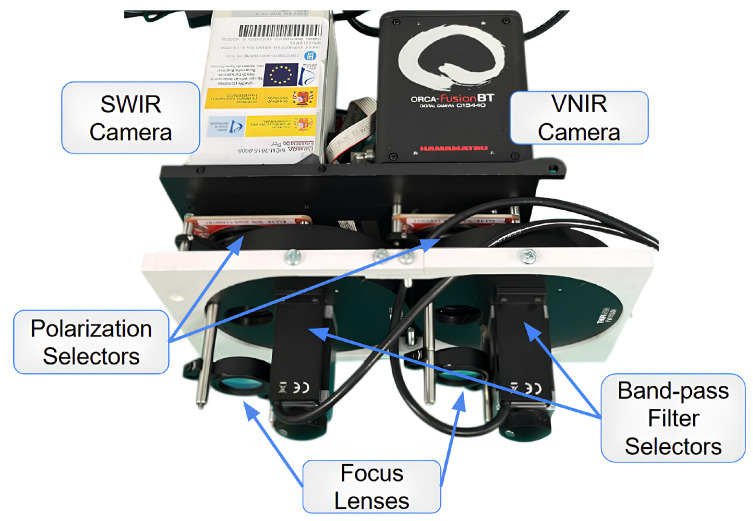
Optical enclosure housing the VNIR and SWIR cameras, spectral filters, and polarimetric elements.

**Figure 7:**
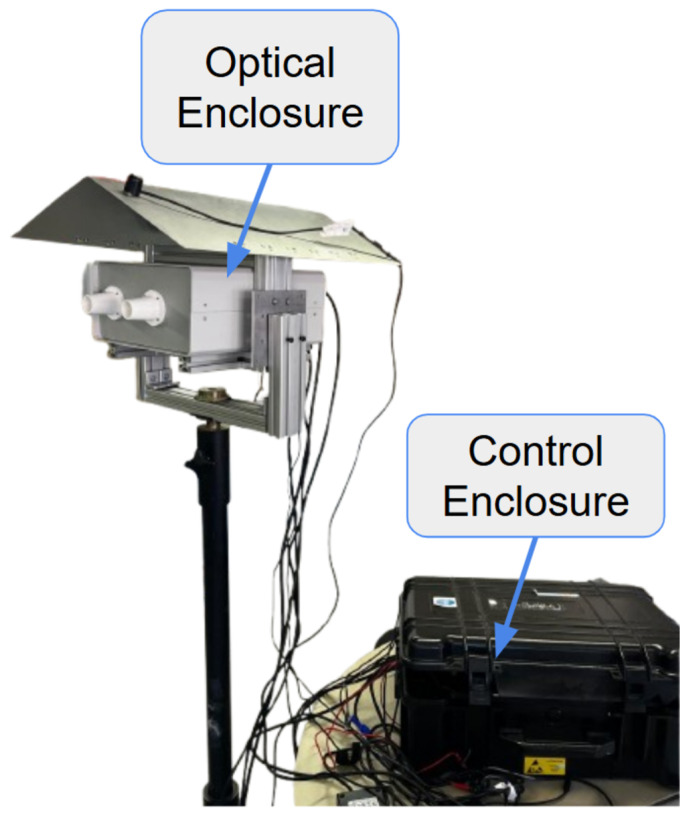
Complete VNIR–SWIR Polarimetric Multispectral Imaging System.

The proposed instrument is a dual-channel Polarimetric Multispectral Imager (PMSI) operating in the VNIR and SWIR spectral ranges (Figure 4). It acquires multispectral cubes composed of 12 discrete spectral bands (430, 490, 550, 630, 700, 750, 930, 1000, 1200, 1300, 1570, 1650 nm) and 4 linear polarization states (V, H, +45°, −45°), producing a total of 48 images per cube. Spectral selection is achieved through motorized filter wheels, while polarization selection is performed by piezoelectric rotation stages. The acquisition process is automated through a nested control loop that sequentially cycles through spectral filters and polarization states, as illustrated in Figure 5. The entire architecture was developed for field-deployable operation. Commercial mechanical components were combined with custom 3D-printed parts specifically designed to ensure mechanical compatibility. To enable practical deployment, two independent enclosures were developed: the *optical enclosure*, housing and protecting all imaging and filtering components, and the *control enclosure*, which contains the drivers and the main computer. The optical enclosure, shown in Figure 6, employs an aluminum frame and is mounted on a dedicated stand to facilitate operation in outdoor environments. The complete system configuration is presented in Figure 7. The system is designed for field use with a total mass of approximately 25 kg, comprising a 10 kg optical head and a 15 kg control unit. The typical time for one complete cube is just over 5 s. The system’s structural rigidity provides stability against moderate winds, and while formal vibration studies have not been conducted, it is designed to withstand standard field handling and transport. To ensure operational stability across varying temperatures and prevent overheating, both the optical and control enclosures are equipped with integrated active ventilation systems.

### 2.1. Passive Sensing Devices

Passive sensing refers to the acquisition of data without emitting any active signal. It relies on sensitive sensors and autonomous devices capable of capturing environmental information for subsequent processing and analysis. A dual-camera architecture (VNIR and SWIR) was prioritized over a single wideband InGaAs sensor to optimize temporal resolution and signal-to-noise ratio. This configuration allows for simultaneous acquisition in both ranges, reducing total measurement time by 50% and supporting the static-scene assumption, which is critical for preventing artifacts in outdoor and underwater environments due to lighting drifts or water motion. Furthermore, dedicated sensors provide superior performance, ensuring higher Quantum Efficiency (QE) and maximizing the signal-to-noise ratio (SNR) across the entire 430–1650 nm range. The VNIR subsystem employs a Hamamatsu ORCA-Fusion BT C15440-20UP (Si CMOS sensor, 2304 × 2304 px, 6.5 μm, USB 3.0), while the SWIR subsystem integrates a Hamamatsu C16090-01 module with an InGaAs sensor G14671-0808W (320 × 256 px, 20 μm, 0.95–1.69 μm, USB 3.1 Gen 1).

### 2.2. Spectral Filtering

For spectral switching, two Thorlabs FW103H/M motorized wheels are employed, with operation managed through dedicated BSC201 drivers. Each wheel hosts up to six 1 inch hard-coated bandpass filters of 10 nm FWHM. Hard-coated bandpass filters use multilayer dielectric interference coatings to transmit a narrow wavelength range while blocking others, ensuring > 95% transmission at the effective central wavelength.

### 2.3. Polarization Filtering

Each spectral subsystem integrates a piezoelectric rotation stage (ELL14K) placed in front of the sensor to control the polarization. The rotation stages are driven by dedicated Elliptec communication and distribution PCBs. Each mount integrates a metallic thin-film wire-grid polarizer (WP25M-UB1, 300 nm–3.2 μm), designed to transmit a defined linear polarization state while attenuating the orthogonal component.

### 2.4. Imaging Optics

Plano-convex lenses featuring AR coatings optimized for their respective spectral bands (AB for VNIR and C for SWIR) are employed to maximize throughput and suppress optical aberrations. To align the images from both sensors, a two-step registration process was used. First, the shared spatial area was co-registered through software centering to account for the different Fields of View (8.5° VNIR vs. 3.6° × 2.9° SWIR). Second, to address the difference in angular resolution, the SWIR images (320×256 px) were upsampled via interpolation to match the higher-resolution VNIR pixel grid (2304×2304 px). To ensure spatial coincidence across all 48 wavelength-polarization channels, sub-pixel registration was performed via median residual alignment. This was validated using calibration targets prior to computing the Stokes reflectance maps.

## 3. Characterization

**Figure 8:**
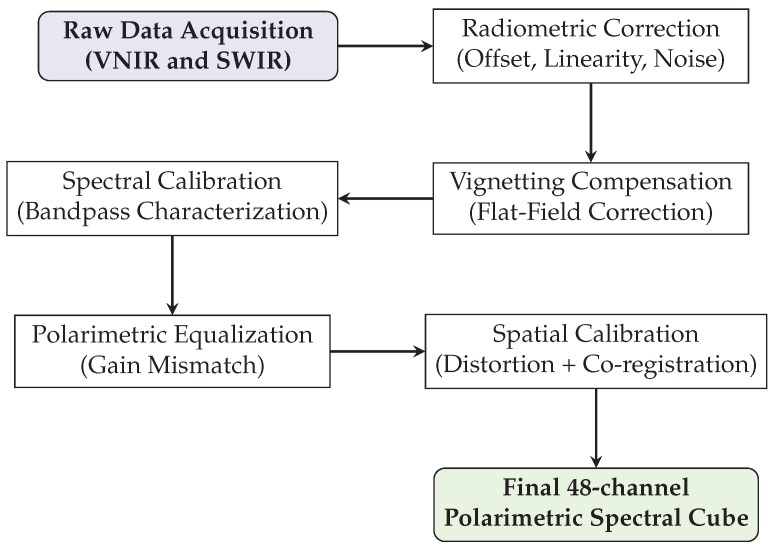
Complete calibration pipeline of the PMSI system: from raw data to a 48-channel polarimetric spectral cube.

Before converting raw data to physical magnitudes, a comprehensive characterization and calibration procedure was performed to ensure the validity of the sensor responses, as summarized in Figure 8.

### 3.1. Functional Tests

**Figure 9:**
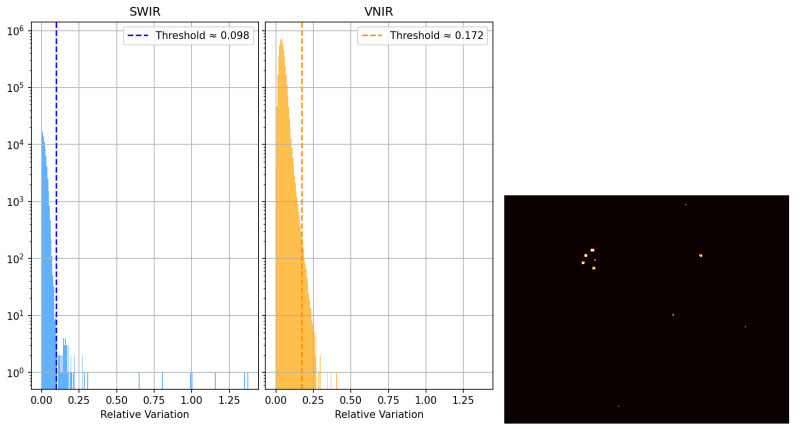
Defective pixel analysis. **Left**: deviation histogram for both sensors; **right**: spatial distribution of defective pixels in the SWIR camera.

The performance tests aimed to validate the linearity, noise level, and uniformity of the imaging system. The Brightness Linearity Test (BLT) was conducted to verify the proportionality between the incident irradiance and the resulting digital output (ADU) across the sensor’s dynamic range. Multiple acquisitions were performed under constant illumination at varying exposure times from 10 μs to 1 s. Both VNIR and SWIR subsystems demonstrated excellent linearity, yielding coefficients of determination ($R^{2}$) exceeding 0.9995 and 0.995, respectively. These results confirm that both sensors maintain consistent linear responses within the operative range. Dark current characterization was performed by acquiring dark frames across various exposure times, maintaining stable thermal conditions within an ISO 8 cleanroom at 21 °C. Both camera sensors have integrated temperature sensors to monitor dark current and offset fluctuations. The SWIR subsystem presented an offset of 13,144 ADU and a dark-current rate of $2.19\times 10^{-3}$ ADU/μs, while the VNIR subsystem showed an offset of 100.4 ADU and a negligible dark current increase below $1\times 10^{-7}$ ADU/μs. The dark frames also revealed a linear relationship between mean intensity and integration time, validating the thermal stability of the detectors. A statistical analysis was performed to detect anomalous behavior by comparing each pixel value to the local mean across homogeneous dark frames, as shown in Figure 9. Defective pixels were identified as those with a dimensionless normalized deviation r>30σ from their neighbors. Here, *r* represents the deviation metric, while μ and σ denote the mean digital value of a pixel and the corresponding spatial standard deviation, respectively, calculated over the ensemble of dark frames.

### 3.2. Optical Corrections

#### 3.2.1. Chromatic Aberration

**Figure 10:**
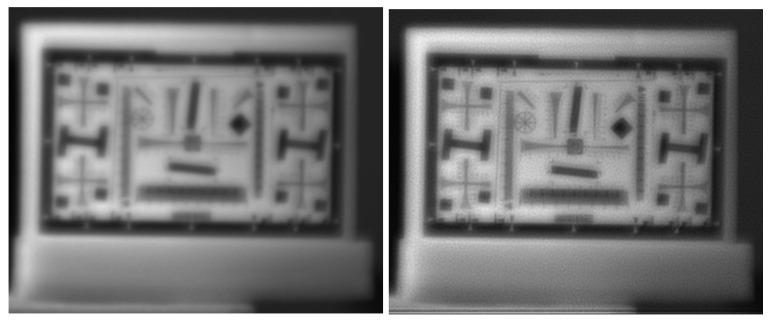
Chromatic aberration correction at 700 nm. **Left**: raw image; **right**: image after Richardson–Lucy deconvolution.

Chromatic aberration (CA) was characterized as a wavelength-dependent defocusing effect inherent to the refractive properties of the imaging lenses. A point-spread function (PSF) was estimated from calibration acquisitions and used to correct each spectral band using the Richardson–Lucy [23] deconvolution algorithm:

(1) $$K^{\left(t+1\right)}=K^{\left(t\right)}\cdot \left(\frac{I}{K^{\left(t\right)}⊗P}⊗P^{*}\right),$$

where $P^{*}$ is the flipped PSF. This iterative correction restored sharpness without requiring lens adjustment [24]. Figure 10 compares a raw and corrected image at 700 nm. Although the deconvolution improves sharpness, residual granularity remains because the scene is not perfectly described by a space-invariant PSF. In addition, bands with large focus offsets cannot be fully corrected with this approach.

#### 3.2.2. Spatial Calibration and Geometric Distortion

To address the spatial aberrations, a geometric calibration was performed using a chessboard pattern. The system’s response was analyzed through a custom automated pipeline that employs a Line Segment Detector (LSD) and k-means clustering to extract and linearize the grid structure.

**Figure 11:**
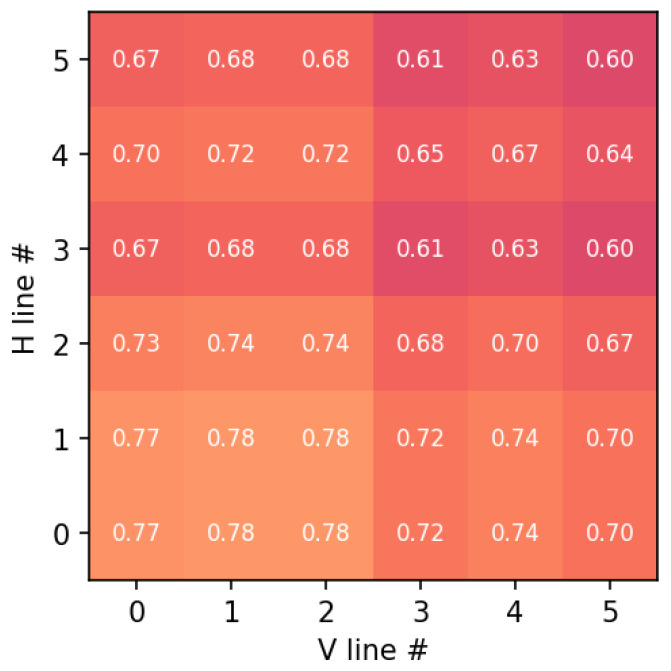
Geometric error map across the 6×6 grid intersections. Each cell corresponds to a grid intersection, where the numerical value indicates the normalized geometric error combining parallelism deviation and orthogonality error. The color scale represents the magnitude of the error, with lighter colors indicating higher error values.

The analysis of the detected segments yielded the following conclusions:

- Parallelism and Orthogonality: The angular deviation between parallel lines remained below 0.5°, and the orthogonality deviation at grid intersections was under 0.8°. These results are summarized in the composite error map in Figure 11.
- Co-registration and Resampling: To compensate for the inherent parallax and the different pixel pitches of the dual-sensor architecture, a sub-pixel co-registration was implemented. Specifically, SWIR frames were mapped to the VNIR reference grid via geometric calibration and sub-pixel co-registration. Registration accuracy was evaluated on calibration targets. Residual misregistration may have remained at high-contrast edges and was accounted for in the uncertainty analysis.

#### 3.2.3. Optical Vignetting and Flat-Field Correction

**Figure 12:**
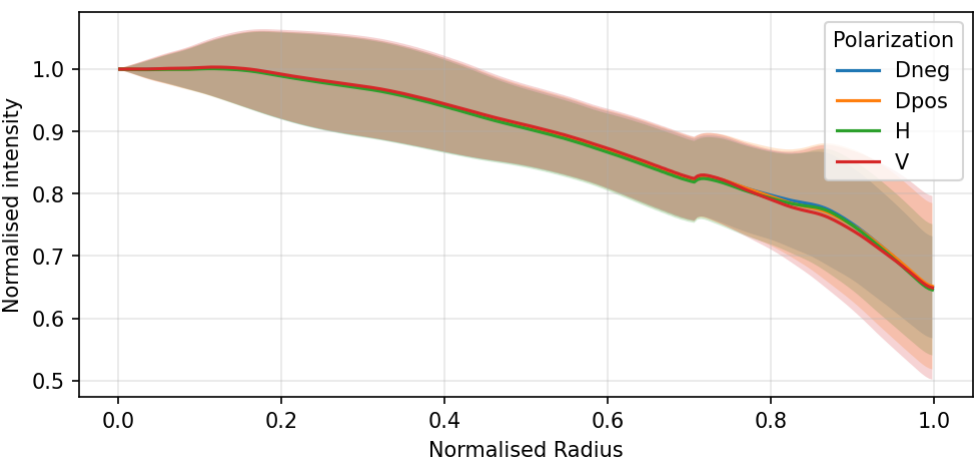
Optical vignetting characterization: normalized intensity profiles as a function of the normalized radius for different polarization states (Dneg, Dpos, H, V). Solid lines represent the mean intensity profiles, while the shaded regions indicate the corresponding standard deviation across the sampled pixels at each radial position.

**Figure 13:**
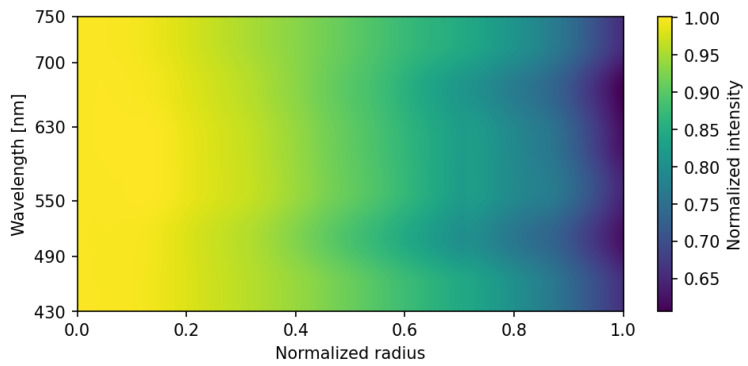
2D intensity distribution across the VNIR spectral range (430–750 nm). The normalized intensity attenuation from the center toward the edge of the field of view (normalized radius) is illustrated, highlighting the wavelength-dependent nature of the optical vignetting in the VNIR channel.

Peripheral illumination roll-off was characterized by analyzing flat-field images of a uniform diffusive white surface. The VNIR subsystem exhibited a radial intensity decrease toward the edge of the field of view, as illustrated by the radial profiles (Figure 12) and the wavelength-dependent intensity map (Figure 13).

This effect was found to be independent of the polarization state (Figure 12) but slightly dependent on wavelength. Conversely, the SWIR subsystem presented negligible vignetting due to the larger image circle of the SWIR optics relative to the sensor size. To ensure spatial uniformity, a pixel-wise flat-field normalization was integrated into the calibration pipeline, effectively neutralizing these systematic attenuations before polarimetric reconstruction.

### 3.3. Polarimetric Spectral Response

Each optical and sensing component contributes a wavelength-dependent transmittance and quantum efficiency to the overall system response. The overall theoretical spectral response, S(λ), was modeled by integrating the filter transmissivity, lens transmittance, and the sensor’s quantum efficiency. The effective wavelength and bandwidth were computed as

(2) $$\langle \lambda \rangle =\frac{\int \lambda S(\lambda )\;d\lambda }{\int S(\lambda )\;d\lambda }.$$

**Figure 14:**
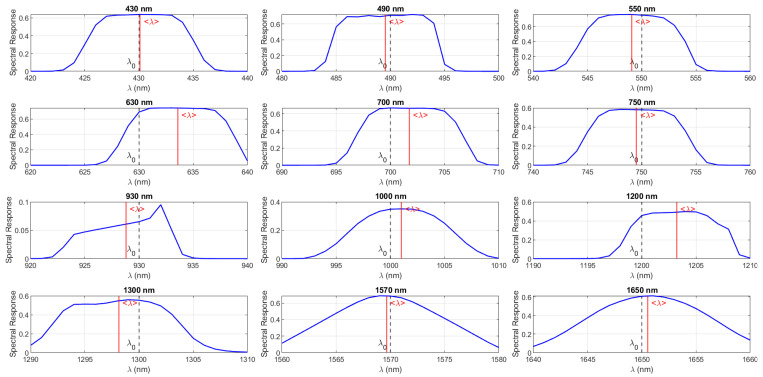
Measured spectral response of the 12 interference filters integrated into the PMSI system. The blue curves indicate the transmittance profile, dashed gray lines ($\lambda_{0}$) represent the nominal center wavelength, and solid red lines (〈λ〉) denote the effective centroid wavelength.

Table 1 summarizes the polarimetric spectral performance of the system, while the corresponding spectral responses of the filters are shown in Figure 14. The effective central wavelengths are generally close to their nominal design values, with small deviations observed at 630 nm and 1200 nm. The calibrated central wavelength 〈λ〉 is computed from the theoretical system spectral response S(λ). The difference between the theoretical and the effective wavelength is defined as Δλ. The Polarization Extinction Ratio (PER) is wavelength-dependent and, according to the manufacturer specifications, sufficient polarization orthogonality is maintained across all operational bands. However, it is acknowledged that the effective PER in the assembled system may be slightly reduced due to practical factors such as minor alignment tolerances, spatial non-uniformity, or fringe effects. To mitigate these effects, channel equalization is applied during calibration, and their contribution is considered within the overall system error budget. The system efficiency varies significantly with wavelength, ranging from 9.5% at 930 nm to 76.27% at 550 nm.

In summary, the characterization campaign validated the performance of both imaging subsystems, confirming linear radiometric response, minimal noise levels, and a well-defined optical throughput across the twelve spectral bands and four polarization states.

### 3.4. Underwater Radiative-Transfer Considerations

**Figure 15:**
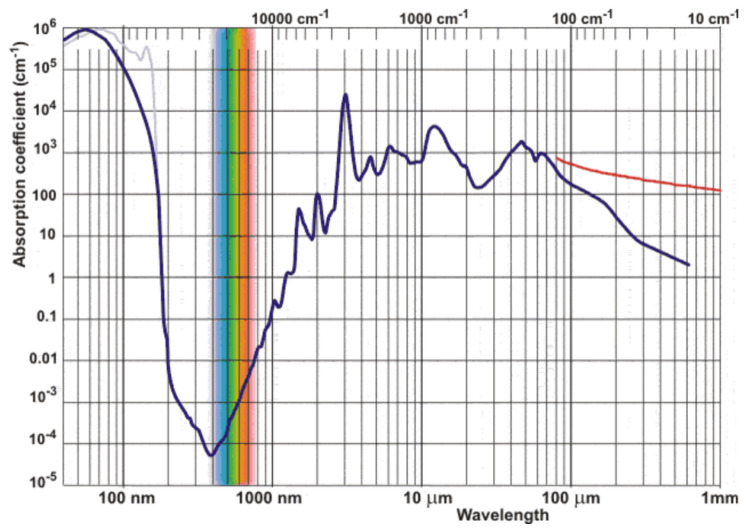
Spectral absorption coefficient of water as a function of wavelength. The colored vertical band highlights the visible (VNIR) spectral region. Low attenuation in the VNIR and strong absorption in the SWIR constrain underwater optical penetration [14].

Light propagation underwater is governed by the combined effects of absorption and scattering, which determine the wavelength-dependent attenuation of radiance. For the spectral range of interest, the radiative-transfer equation can be locally approximated by the Beer–Lambert law:

$$I\left(z,\lambda \right)=I_{0}\left(\lambda \right)\;e^{-\alpha (\lambda )\;z},$$

where *z* is depth and α(λ)=a(λ)+b(λ) is the sum of the absorption a(λ) and scattering b(λ) coefficients. The absorption coefficient of pure water increases by several orders of magnitude from the VNIR to the SWIR region, as shown in Figure 15, yielding attenuation lengths below a few millimeters above approximately 1000 nm. The underwater illumination–viewing geometry and the refraction at the air–water interface also influence the received radiance through Fresnel transmission effects. While these parameters were not fully controlled in the present experiment, the empirical trends reported here agree with classical radiative-transfer predictions and with recent underwater multispectral imaging studies.

### 3.5. System Error Budget

System characterization reveals several bounded instrumental sources of uncertainty that contribute to the overall error budget. The camera response was verified to be linear, with calibrated gain and offset; however, the SWIR sensor exhibits temperature-dependent offset drift and increased dark counts. Residual chromatic aberrations and channel misregistration introduce wavelength-dependent spatial errors, while geometric distortion and vignetting can be effectively corrected. The system spectral response is based on manufacturer specifications rather than in situ spectral characterization. Finally, the polarimetric subsystem is affected by finite polarization extinction ratio and limited angular repeatability of the rotation stage, resulting in residual polarimetric uncertainty, assessed by propagating the extinction leakage ϵ=1/PER into the Stokes equations.

## 4. Data Processing

### 4.1. Reflectance and Polarimetric Separability of Dry Plastics

After system characterization, the next step was to evaluate its ability to identify materials under controlled dry conditions. A dedicated experiment was designed to acquire polarimetric multispectral cubes of a plastic matrix composed of various polymers with distinct optical and chemical properties.

#### 4.1.1. Experimental Setup

**Figure 16:**
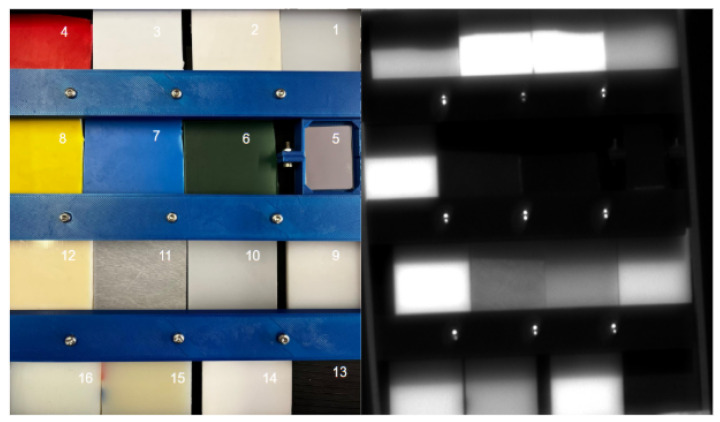
Plastic sample matrix used for reflectance measurements. The numbered samples correspond to the materials listed in the text. **Left**: RGB composite; **right**: monochromatic image at 630 nm and horizontal polarization.

A matrix composed of sixteen 2 × 2 inch samples was prepared, including: (1) Ultra-High-Molecular-Weight polyethylene (UHMW), (2) polyester, (3) polystyrene (PS), (4) red PS, (5) silver mirror, (6) green PS, (7) blue PS, (8) yellow PS, (9) high-density polyethylene (HDPE), (10) low-density polyethylene (LDPE), (11) Rexolite, (12) acrylonitrile butadiene styrene (ABS), (13) modified polyphenylene oxide (Noryl PPO), (14) Delrin acetal, (15) extruded nylon, and (16) cast nylon. Figure 16 shows the RGB overview and a representative monochromatic image.

#### 4.1.2. Relative Reflectance Estimation

**Figure 17:**
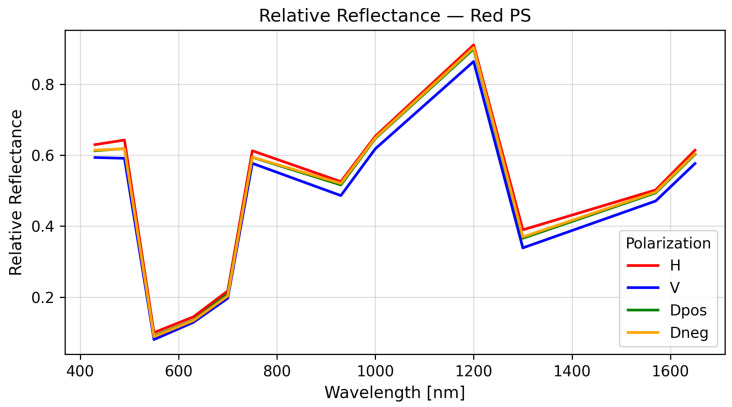
Relative polarimetric spectral signature of red polystyrene.

**Figure 18:**
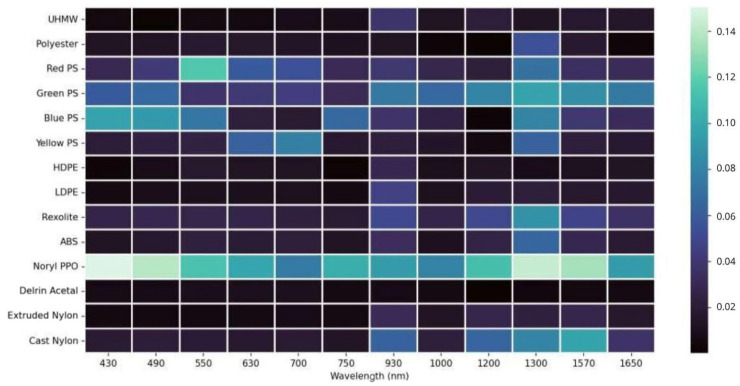
Band-dependent degree of linear polarization for the selected plastic materials.

As a first step toward polymer identification, we compared the digital numbers of each region of interest (ROI) with those from the reference mirror. The relative reflectance ρ(i,T) is expressed as Equation (3):

(3) $$\rho \left(i,T\right)=\frac{D_{i,T}-D_{\mathrm{offset}}}{D_{i,\mathrm{mirror}}-D_{\mathrm{offset}}},$$

where $D_{i,T}$ is the pixel intensity of the target material, $D_{i,\mathrm{mirror}}$ the corresponding intensity from the reference, and $D_{\mathrm{offset}}$ the dark-current baseline. This normalization provides an initial database of polarimetric spectral signatures (PSSs) without requiring absolute irradiance calibration. A representative example of a polarimetric spectral signature is shown in Figure 17. The degree of linear polarization (DoLP) for each material was computed using

(4) $$\mathrm{DoLP}=\frac{\sqrt{Q^{2}+U^{2}}}{I},$$

where *I* is the total intensity, and *Q* and *U* are the Stokes parameters associated with horizontal–vertical and diagonal–antidiagonal linear polarizations, respectively. The wavelength-dependent DoLP behavior for the analyzed materials is presented in Figure 18. Materials exhibited distinct DoLP behavior depending on their microstructure and surface roughness, with polystyrene derivatives showing strong polarization sensitivity, while polyester and polyethylene families remained largely unpolarized.

The main disadvantage of this method is that it requires a calibrated reference target (mirror) within the scene for every acquisition. Nevertheless, this first iteration confirms that materials exhibit measurable polarimetric behavior, motivating the use of polarization features to improve separability.

#### 4.1.3. Polarimetric Channel Equalization

Assuming that linear polarization states are uniformly affected by atmospheric transmission, the intrinsic polarization-dependent transmission of the system remains to be further investigated. Polarimetric calibration was conducted using an unpolarized light source to compensate for residual gain imbalances between the different channels. The correction factors $g_{i}$ were defined as Equation (5):

(5) $$g_{i}=\frac{{\bar{I}}_{\mathrm{ref}}}{I_{\mathrm{ref},i}},\;i\in \left\{V,H,D^{+},D^{-}\right\}.$$

**Figure 19:**
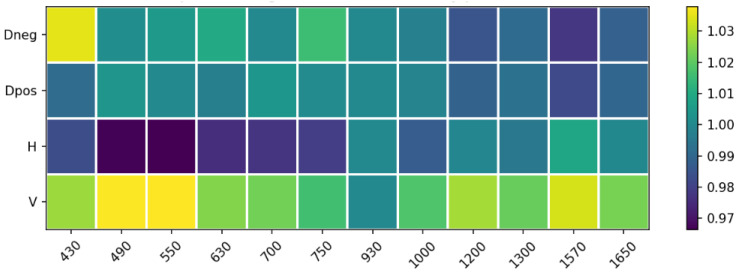
Polarimetric channel gain correction as a function of wavelength.

The application of these gain factors ensures a uniform response across all polarization channels for unpolarized light, effectively reducing systematic errors to below 3% throughout the spectral range, as shown in Figure 19.

#### 4.1.4. Absolute Reflectance Calibration

For outdoor acquisitions, absolute reflectance was calibrated against the solar AM1.5 spectrum, with local atmospheric corrections implemented using MODTRAN model [25]. All experimental datasets were acquired under illumination conditions broadly consistent with the AM1.5 solar spectrum, enabling the use of MODTRAN simulations as a physically grounded reference. In dry experiments, plastic samples were positioned approximately normal to the incident solar illumination. Absolute reflectance maps were computed by applying the calibrated conversion factors to each polarization channel and spectral band, followed by Stokes parameter computation and DoLP estimation.

The pyranometer readings provided a reference irradiance $E_{\lambda }\left(t\right)$, allowing conversion from measured radiance as ADU to reflectance. $D_{\lambda ,P}\left(x,y,t\right)$ is the raw camera measurement (in ADU) at pixel (x,y), wavelength λ and polarization state *P*, and $D_{offset}\left(t\right)$ is the temporal bias or dark offset. $G_{\mathrm{DN}\to e^{-}}$ converts ADU to electrons. On the right side of the equality, the detected electrons are expressed as the result of optical radiance: $A_{\mathrm{px}}$ is the pixel photosensitive area, Ω is the solid angle corresponding to the pixel field of view, $t_{\mathrm{int}}$ is the integration time, $S_{i}\left(\lambda \right)$ is the spectral response of channel *i*, $L_{\lambda ,P}\left(x,y,t\right)$ is the incoming spectral radiance, and the factor λ/(hc) converts radiance into photon flux using Planck’s constant *h* and the speed of light *c*.

(6) $$N_{e^{-},\lambda ,P}\left(x,y,t\right)=G_{\mathrm{DN}\to e^{-}}\left[D_{\lambda ,P}\left(x,y,t\right)-D_{offset}\left(t\right)\right]=A_{\mathrm{px}}\;\Omega \;t_{\mathrm{int}}\int S_{i}\left(\lambda \right)\;L_{\lambda ,P}\left(x,y,t\right)\;\frac{\lambda }{hc}\;d\lambda .$$

${\langle L\rangle }_{\lambda ,P}$ is the band-averaged radiance reaching the sensor, obtained from the detected electrons while accounting for pixel area, solid angle and integration time. ${\langle E\rangle }_{\lambda }$ is the band-averaged irradiance reaching the target, obtained by spectrally weighting the illumination $E_{\lambda }\left(t\right)$ using the same instrumental response $S_{i}\left(\lambda \right)$. The absolute reflectance $\rho_{\lambda ,P}$ is computed as the ratio between radiance and irradiance, scaled by π under the Lambertian assumption.

(7) $$\begin{array}{cc} {\langle L\rangle }_{\lambda ,P} & =\frac{N_{e^{-},\lambda ,P}}{A_{\mathrm{px}}\;\Omega \;t_{\mathrm{int}}}\cdot \frac{hc}{\int S_{i}\left(\lambda \right)\lambda \;d\lambda },\\ {\langle E\rangle }_{\lambda } & =\frac{\int S_{i}\left(\lambda \right)\;E_{\lambda }\;d\lambda }{\int S_{i}\left(\lambda \right)\;d\lambda },\\ \rho_{\lambda ,P} & =\pi \cdot \frac{{\langle L\rangle }_{\lambda ,P}}{{\langle E\rangle }_{\lambda }}. \end{array}$$

**Figure 20:**
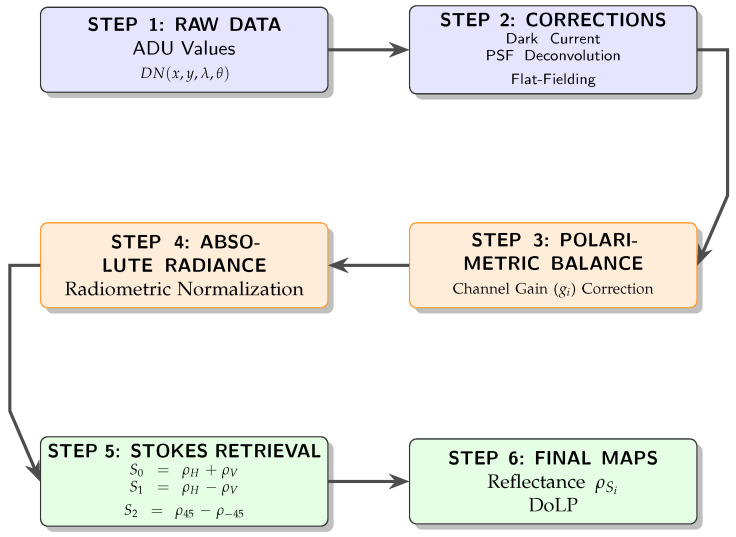
Flowchart of the PMSI data processing pipeline: from raw ADU values to DoLP reflectance maps. Linear polarization measurements correspond to vertical (V), horizontal (H), +45° (D^+^), and −45° (D^−^) analyzer orientations.

For any wavelength λ, the polarization associated reflectance is written as $\rho_{\lambda ,P}\equiv \rho_{P}$. The overall processing workflow, from raw ADU measurements to final reflectance and DoLP maps, is summarized in Figure 20. The four measured reflectances ($\rho_{V},\;\rho_{H},\;\rho_{D^{+}},\;\rho_{D^{-}}$) map to the Stokes reflectance components through Equation (8):

(8) $$\left[\begin{array}{c} \rho_{S_{0}}\\ \rho_{S_{1}}\\ \rho_{S_{2}} \end{array}\right]=\left[\begin{array}{cccc} 1 & 1 & 0 & 0\\ -1 & 1 & 0 & 0\\ 0 & 0 & 1 & -1 \end{array}\right]\left[\begin{array}{c} \rho_{V}\\ \rho_{H}\\ \rho_{D^{+}}\\ \rho_{D^{-}} \end{array}\right]$$

The degree of linear polarization (DoLP) is then obtained as Equation (9):

(9) $$\mathrm{DoLP}=\frac{\sqrt{\rho_{S_{1}}^{2}+\rho_{S_{2}}^{2}}}{\rho_{S_{0}}}.$$

**Figure 21:**
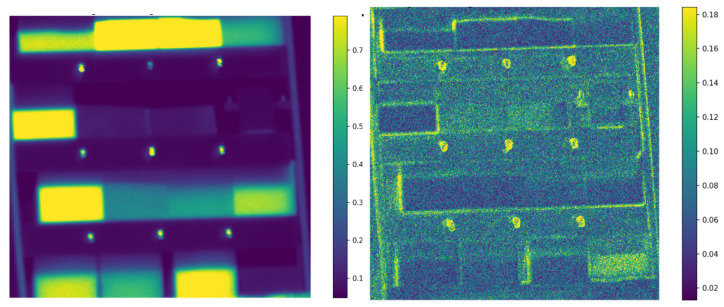
Stokes reflectance maps at 430 nm. **Left**: $S_{0}$; **right**: $S_{1}$.

**Figure 22:**
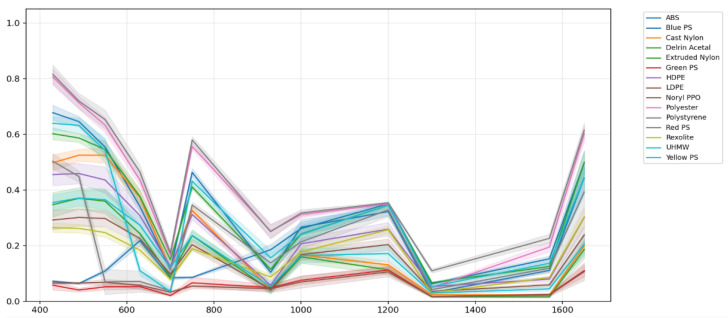
First Stokes reflectance spectral signatures of the selected polymers (430–1650 nm). Curves show mean ROI reflectance with ±σ uncertainty.

Stokes reflectance maps are computed for each spectral band, transforming the 48 polarization–wavelength ADU images into 36 Stokes-wavelength absolute reflectance maps (ρ). New ROIs are selected to obtain each material absolute Stokes Spectral Signatures. Figure 21 shows these maps for the first and second Stokes reflectance components at 430nm. Figure 22 shows the mean and standard deviation of the first Stokes reflectance component of the selected polymers.

### 4.2. Material Separability Analysis

Spectral separability between materials was quantified using the Euclidean distance in the 12-dimensional wavelength space:

(10) $$D_{E}\left(x,y\right)=\sqrt{\sum_{i=1}^{d}{\left(x_{i}-y_{i}\right)}^{2}}.$$

**Figure 23:**
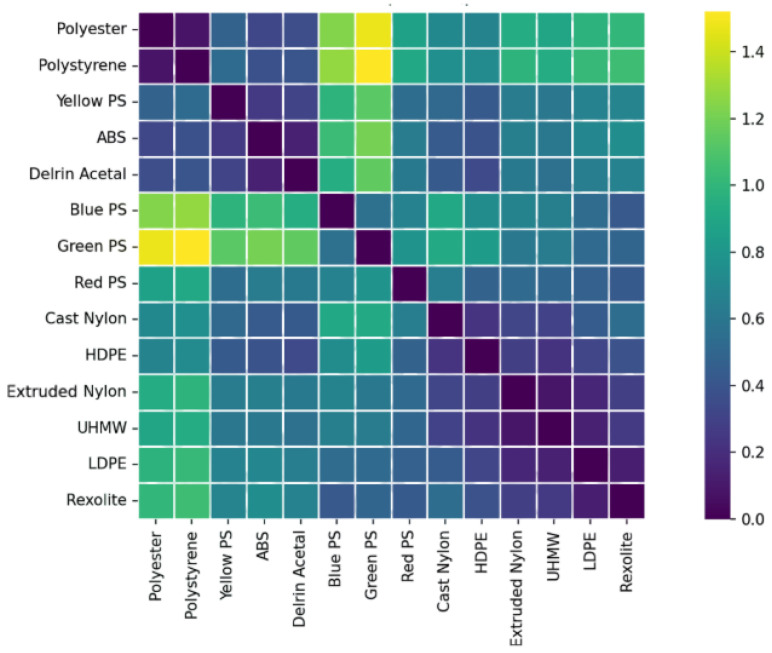
Spectral separability between materials computed from $S_{0}$ reflectance.

**Figure 24:**
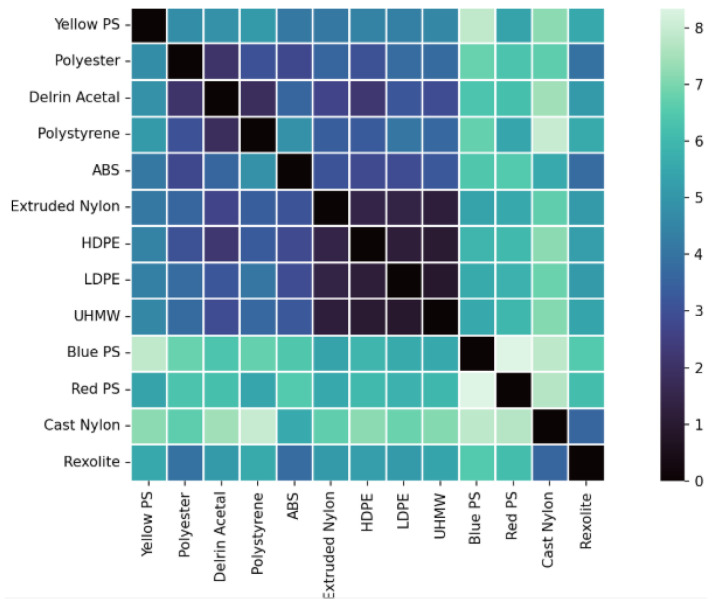
Spectral separability between materials computed from the degree of linear polarization (DoLP).

Euclidean distance was selected as a direct and interpretable measure of separability, given that all spectral signatures are fully band-aligned and radiometrically calibrated. Two magnitudes were analyzed: the total Stokes reflectance ($S_{0}$) and the normalized degree of linear polarization (DoLP). Inter-material separability was quantified using pairwise Mahalanobis distances computed in three feature spaces: intensity-only reflectance, DoLP-only, and combined reflectance–DoLP. The separability results based on $S_{0}$ reflectance are shown in Figure 23, while the separability derived from DoLP is presented in Figure 24.

**Figure 25:**
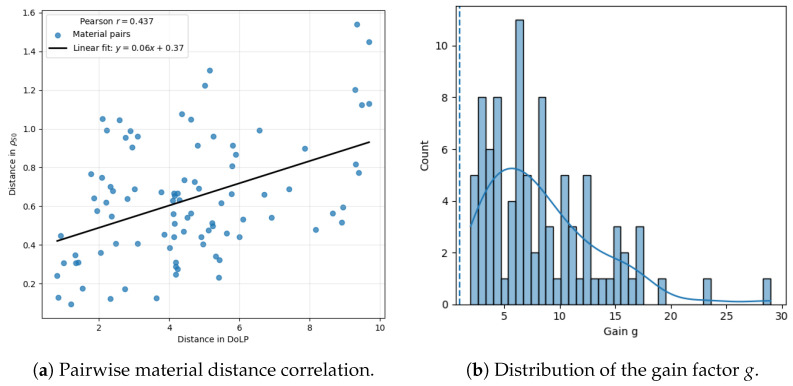
Comparison between material separability based on $S_{0}$ reflectance and DoLP. Left: pairwise material distance correlation; right: distribution of the gain factor *g*. The dotted line indicates unit gain (g=1), corresponding to equal separability between both metrics.

Figure 25 shows the gain factor *g*, defined for each material pair as the ratio between Euclidean separability distances computed from DoLP and from $S_{0}$ reflectance. The resulting distribution of *g* is right-skewed, with a mean of 8.374 (95% CI: [7.363,9.468]) exceeding the mean of 6.983 (95% CI: [6.354,8.473]). This gap, alongside an inter-quartile range of 4.376–10.953, shows that material pairs show moderate improvement. Despite a moderate Pearson correlation (r=0.437) between both distance metrics, the DoLP-based approach consistently yields significantly higher separability than intensity-only reflectance. DoLP exhibits substantially larger relative variability than intensity-only reflectance. Across all materials and bands, the mean coefficient of variation is ${\mathrm{CV}}_{\rho_{S0}}=0.082$ (IQR: 0.048–0.113) versus ${\mathrm{CV}}_{DoLP}=0.458$ (IQR: 0.265–0.506), a ∼5.6× higher relative variability for DoLP. This behavior is consistent with $S_{1}$ and $S_{2}$ being strongly sensitive to surface properties. The higher CV in DoLP (0.458) compared to reflectance (0.082) is primarily attributed to the high sensitivity of polarization to surface geometry and facet orientation. No temporal averaging was used to preserve the instantaneous response. Spatial registration was performed at the pixel level.

Separability maps obtained reveal that $S_{0}$ and DoLP provide different information. The $S_{0}$ matrix offers the separability in total reflectance intensity, and through this some trends can be observed.

- High separability (>1) is only observed when comparing white materials (polyester, polystyrene) with colored and dark plastics.
- Rexolite, Nylon, ABS, and Delrin Acetal present similar distance values between most materials (∼0.6).
- The polyethylene family (LDPE, HDPE, UHMW) still shows low separability (∼0.2), consistent with the study using relative reflectance.

The conclusion is that through only $S_{0}$ values, robust differentiation for materials of different colors can be done but not for materials of the same composition (chemical similarity) or with a lack of specific pigmentation.

Through the DoLP matrix, a much higher separability absolute value between all objects is observed. Noryl PPO, which already showed a substantial polarimetric effect in Figure 18, was not taken into account for this matrix as it had large (over 15) separability with all materials. Again, three different behaviors can be extracted, depending on the materials:

- Large separability (>6) is observed between pigmented materials of different chemical compositions (e.g., Red PS and Blue PS).
- Moderate separability (∼4) is found for most materials with different chemical compositions (e.g., Delrin Acetal, polyester, ABS, Rexolite, …).
- The three polyethylene materials still show low separability, with the lowest separability values (∼1) in the matrix. This highlights an intrinsic limitation of DoLP-based discrimination when both spectral and polarimetric contrast are inherently low.

Studying materials through DoLP enhances the relative distance between these materials, even though very similar materials still have difficulties in separability. This study allows us to construct a database, consisting of the reflectance associated with the three first Stokes parameters for each band of each material, which can be used as the base for different applications.

### 4.3. Spectral Unmixing Methods

Spectral unmixing was employed to assess the separability of materials in the multispectral and polarimetric feature space. Endmember extraction was performed using established convex-geometry-based algorithms, including the Vertex Component Analysis (VCA) method [26], the N-FINDR algorithm [27], and the Pixel Purity Index (PPI) approach [28]. These methods aim to identify spectrally pure pixels by exploiting the geometric properties of multispectral data in a reduced-dimensionality space.

For completeness, we note that VCA assumes the presence of at least one pure pixel per endmember and operates by iteratively projecting the data onto randomly generated directions [26]. N-FINDR maximizes the volume of a simplex defined by candidate endmembers [27], while PPI identifies extreme pixels through repeated random projections [28]. A broader overview of spectral unmixing techniques and their assumptions can be found in [29,30].

## 5. Material Information Recovery

### 5.1. Spectral Unmixing

Once the PSS database is constructed, algorithms are required to recover material information in more realistic scenes. Spectral unmixing methods (SUM) were therefore used for this purpose.

#### 5.1.1. Linear Mixing Model

The reflectance of each pixel was modeled as a linear combination of *p* material spectra (endmembers):

(11) X=MS+E,

where $X\in R^{L\times N}$ is the multispectral cube with *L* spectral bands and *N* pixels, $M\in R^{L\times p}$ contains the endmember signatures, $S\in R^{p\times N}$ their abundance fractions, and E the residuals. Each endmember corresponds to a polymer signature extracted from the reflectance–polarimetric database.

#### 5.1.2. Endmember Extraction Methods

Three endmember extraction algorithms were evaluated to identify the most representative spectral signatures in the dataset [29,31,32]: Vertex Component Analysis (VCA), N-FINDR, and Pixel Purity Index (PPI). The number of endmembers *p* was estimated using HySime and Virtual Dimensionality (VD), which determine the intrinsic dimensionality of the data by maximizing SNR and testing eigenvalue significance. Due to the limited number of spectral bands (L=12) and the resulting low intrinsic dimensionality, the number of endmembers was estimated using HySime on four spatial subregions per image. The estimates were consistent across subregions (typically k≈4), and PCA-explained variance saturation after the first 2–4 components further supports this choice. Therefore, p=4 was selected as a conservative and stable value. Unmixing was performed using VCA, PPI, and N-FINDR with the same value of *p* and the referenced configuration. VCA selects endmembers through a fixed *p*-step procedure. PPI was run with a fixed number of random projections (5000 skewers). N-FINDR was executed with a maximum of 3p iterations and terminated early when the simplex volume no longer increased. All randomized steps were made reproducible by fixing the MATLAB random seeds for both uniform and Gaussian generators prior to execution [31].

#### 5.1.3. Performance Evaluation Metrics

Recovered endmembers $M_{\mathrm{est}}$ were compared with the reference database $M_{\mathrm{ref}}$ through four metrics:

1. Root Mean Square Error (RMSE):
  (12) $$\mathrm{RMSE}=\sqrt{\frac{1}{L}\sum_{i=1}^{L}{\left(M_{\mathrm{ref},i}-M_{\mathrm{est},i}\right)}^{2}}$$
2. Spectral Angle Mapper (SAM) [19]:
  (13) $$\mathrm{SAM}=\cos^{-1}\;\left(\frac{M_{\mathrm{ref}}\cdot M_{\mathrm{est}}}{∥M_{\mathrm{ref}}∥\;∥M_{\mathrm{est}}∥}\right)$$
3. Spectral Information Divergence (SID) [33]:
  (14) $$\mathrm{SID}=\sum_{i}\left(p_{i}\log\frac{p_{i}}{q_{i}}+q_{i}\log\frac{q_{i}}{p_{i}}\right)$$
4. Execution Time: total processing time required to estimate endmembers and abundance maps.

#### 5.1.4. Results and Discussion

**Figure 26:**
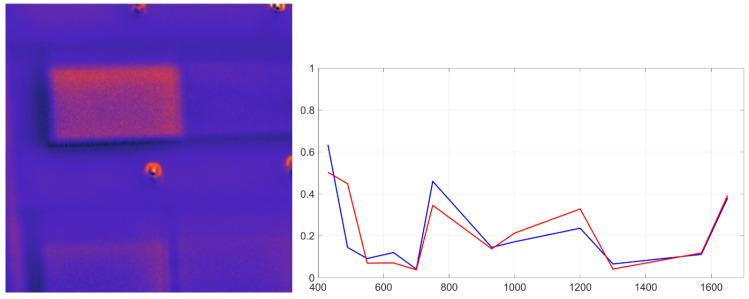
Spectral unmixing result for red polystyrene using VCA. **Left**: abundance map; **right**: reference (red) and recovered (blue) spectral signatures.

**Figure 27:**
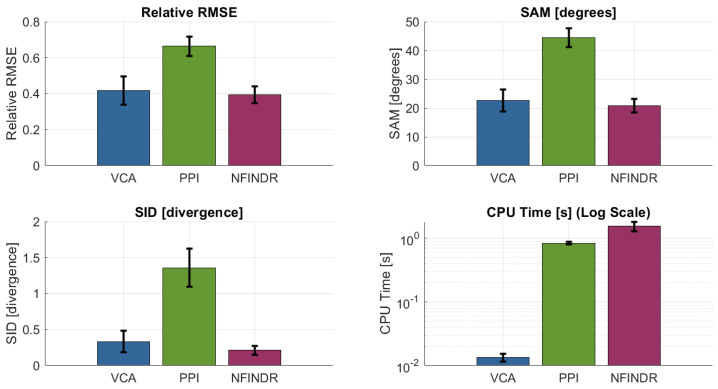
SUM performance metrics. VCA and N-FINDR show similar results, but VCA converges much faster.

All methods were applied to the multispectral cubes of the plastic matrix. VCA provided a favorable trade-off between computational complexity and accuracy, consistently identifying endmembers that matched laboratory-measured PSS signatures. VCA correctly localizes materials such as red polystyrene (Figure 26). The overall performance comparison of the evaluated methods is shown in Figure 27.

Although N-FINDR achieved similar reconstruction accuracy, it required two orders of magnitude longer processing time due to its combinatorial volumetric search. PPI was faster but yielded lower spectral fidelity and was more sensitive to noise. VCA provided a favorable trade-off between computational complexity and accuracy, consistently identifying endmembers that matched laboratory-measured PSS signatures.

**Figure 28:**
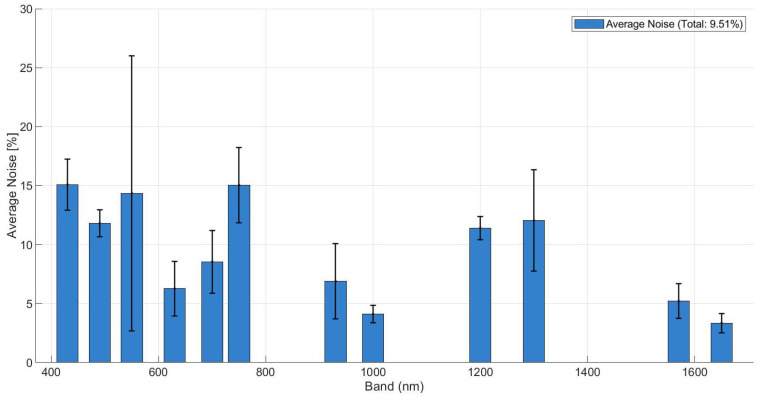
Estimated residual noise level of the $\rho_{S0}$ signal per spectral band, obtained using the HySime algorithm.

To further validate the robustness of the unmixing process, an uncertainty analysis was conducted. The intrinsic noise floor of the $\rho_{S0}$ signal was estimated using the HySime algorithm, revealing a mean residual noise of approximately 5–15% across the spectral bands, as shown in Figure 28. Despite this high-noise environment, the unmixing metrics remained stable, confirming the algorithms’ resilience. However, the anticipated performance gain from the degree of linear polarization (DoLP) was partially offset by its inherently higher noise levels and the current limitations of our discrete 12-band spectral sampling. Although polarimetric signatures ($S_{1},S_{2}$) provide a theoretical advantage in material separability by capturing surface roughness features, this gain is currently comparable in magnitude to the experimental noise floor, particularly when considering material-dependent background effects, as similarly observed in fluorescence-based material discrimination studies [34].

Not all materials were detected in the scene. This is expected since the spectral library contains only 12 bands and 16 materials, whereas these algorithms were originally designed for datasets with hundreds of bands and endmembers. Two consequences arise: (i) weak signatures (dark materials) may be absorbed into other pixels, and (ii) misclassification can occur when materials still have similar polarimetric spectral signatures. Increasing the number of spectral bands would improve detection, coupled to extension of the database, which could contain hundreds of Stokes Spectral Signatures. Overall, combining VCA with the spectral–polarimetric database enables reliable unmixing and spatial recovery of polymers, demonstrating that even with few discrete bands, multispectral unmixing is feasible when guided by a calibrated reflectance polarimetric reference library.

### 5.2. Submerged Plastic Detection

An underwater detectability study was conducted under natural illumination to assess practical depth and wavelength constraints for submerged plastic detection. The detection of plastics in aquatic environments is strongly affected by the optical properties of water, which attenuates incident light depending on wavelength. Understanding how spectral and polarimetric information behaves underwater is essential for evaluating the system’s applicability to realistic environmental monitoring scenarios [35]. The same 4 × 4 plastic matrix used for the dry tests was placed flat freely floating on a freshwater tank under natural illumination. The water tank was kept static to prevent the formation of standing waves, which could alter the effective incidence plane of the illumination and introduce uncontrolled variations in both intensity and polarization. Measurements were acquired at five immersion depths (0, 4, 8, 12, and 20 cm) to capture the complete range of optical attenuation. The 0 cm depth provides a baseline reference for dry materials, and 4, 8, and 12 cm serve as transition zones to observe signal degradation. Finally, 20 cm represents the maximum possible immersion depth of the water tank. Underwater experiments were designed to address different objectives and were therefore evaluated qualitatively as calibrated intensity images (corrected for sensor bias and dark current but prior to Stokes vector transformation) already provide meaningful insights into material detectability. The results indicate a strong wavelength dependence. However, it must be noted that polarimetric contrast can be masked by factors such as residual InGaAs sensor noise, underwater backscattering, and the low Signal-to-Noise Ratio (SNR) caused by strong water absorption in the SWIR bands. Despite these effects, the applied radiometric calibration and dark current correction allow for a clear observation of the polymers’ silhouettes and their relative contrast against the background.

#### 5.2.1. Loss of Penetration Capability at SWIR Bands

Due to the exponential absorption coefficient increase, as soon as materials are slightly submerged, they are no longer detectable when studying SWIR wavelengths. SWIR bands beyond 1000 nm exhibited rapid attenuation, limiting reliable detection to depths of approximately 4 cm in the tested conditions. Even the images taken at 4 cm depth show no separability as water behaves as an opaque wall. Only at 930 nm the matricial form of the plastic testbench can be observed but in no case used for plastic recovery. It can be concluded that SWIR polarimetric imaging improves separability in dry materials, but as soon as water is involved, it completely loses capabilities.

#### 5.2.2. Submerged Plastic Identification Using VNIR Wavelengths

**Figure 29:**
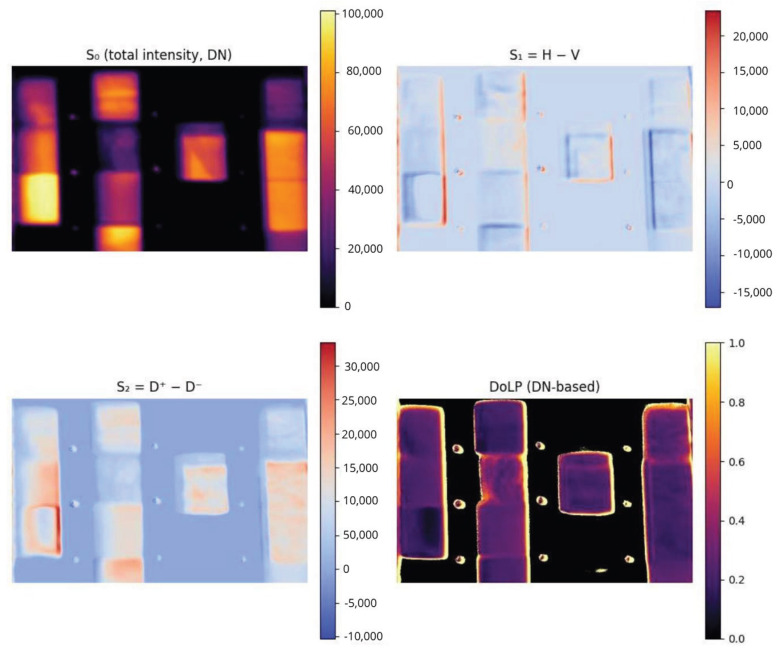
Underwater polarimetric imaging of plastic samples at 430 nm and 20 cm depths.

For comparison with framework [17], the matrices related to total incident intensity, and the differences H-V and (+45°)−(−45°) ($D^{+}-D^{-}$) and DoLP at Digital Number (DN) were computed for each band, for each depth. VNIR bands in the 430–550 nm range preserved detectability up to approximately 20 cm, with shorter VNIR wavelengths generally outperforming longer VNIR bands due to lower absorption and scattering losses. As expected, the images where water absorption is less significant are the ones with the best results (430, 490, and 550 nm). Even though most materials inside become indistinguishable as a direct consequence of water absorption, this method allows us to observe the shape of the materials and what makes them easily detectable, no matter the depth of the test framework (0–20 cm). We hypothesize that it may be caused by a polarimetric gradient due to irregularities on the material edges [36]. Figure 29 shows how the shape of materials that have sufficient reflectance underwater is highlighted when analyzed through polarimetry. This phenomenon provides a framework for low-cost processing for material detection.

The following conclusions can be extracted from the plastic submersion campaign:

- Water absorption behavior was confirmed as SWIR wavelengths were absorbed, and the best ones were in the range 400–550 nm. The SWIR subsystem, although effective for dry plastic identification, does not perform when plastics are submerged.
- VNIR regions obtain polarimetric optical information that enhances detectability. Polarimetric processing reduced glint-induced false positives by discriminating polarized surface reflections from the partially depolarized light scattered by submerged targets.
- As most of the information on different spectral bands is insufficient, with the information recovered we cannot define a testbench for underwater plastic distinguishability, but it provides a framework of material detectability.
- Measurements are highly dependent on the environmental conditions. Nonetheless, an improvement in detectability should be expected when observing irregular materials since polarimetric characteristics tend to enhance the lateral edges and surface discontinuities.

## 6. Discussion

Under dry conditions, the calibrated reflectance and DoLP measurements showed that polarimetric information provides a statistically significant improvement in discriminability across most polymers. Overall, DoLP enhances inter-material separability relative to intensity-only reflectance, with a mean gain of 6.9 (95% CI: 6.35–8.47) and a higher mean gain of 8.37, reflecting a right-skewed distribution, while only a moderate Pearson correlation (r = 0.44) is observed between intensity and DoLP-based separability.

Polymers with highly similar chemical composition (e.g., HDPE, LDPE, and UHMW) remained difficult to separate, highlighting intrinsic limits imposed by their weak optical anisotropy and by the reduced number of spectral bands.

Moreover, the heavy reliance on theoretical spectral responses and idealized AM1.5 illumination for calibration could undermine the system’s robustness in variable field conditions; consequently, it is essential to evaluate how effectively these results transfer to realistic aquatic environments where non-Lambertian effects and varying sky irradiance predominate. In practice, the standard AM1.5 model does not account for the spectral modulation induced by local atmospheric constituents, such as aerosol optical thickness or fluctuating water vapor, which can distort the down-welling irradiance curve.

Furthermore, the transition from laboratory to aquatic settings introduces complex air–water interface phenomena, including surface glint and depth-dependent attenuation. These factors challenge the assumption of a static illumination source as the light field reaching a submerged target is subject to both Rayleigh and Mie scattering, as well as selective absorption by colored dissolved organic matter (CDOM). Therefore, while the current calibration provides a rigorous baseline, achieving true operational reliability requires a transition toward dynamic radiometric correction methods that can adapt to the inherent optical properties (IOPs) of real-world water bodies.

The underwater experiments revealed strong wavelength-dependent attenuation. SWIR bands lost penetration capability beyond 4 cm, whereas VNIR wavelengths between 430 and 550 nm preserved material detectability down to 20 cm. Polarimetric contrast remained relatively stable underwater and consistently enhanced edge-level detectability of plastic targets under controlled tank conditions. However, this enhancement should be interpreted primarily in terms of detectability rather than material-specific classification.

It should be emphasized that both reflectance and DoLP depend on illumination–viewing geometry and surface roughness. In general, these dependencies are described by the Bidirectional Reflectance Distribution Function (BRDF) and its polarimetric extension (pBRDF). Consequently, the measurements reported in this work correspond to a fixed and repeatable acquisition geometry, meaning that the mirror normalization applied is valid strictly for those specific conditions. For future field deployments, the challenges of variable geometry and changing surface states will necessitate multi-angle calibration and/or comprehensive BRDF/pBRDF modeling, both of which are planned as next steps in our research. Other limitations of the current prototype include reduced SWIR efficiency, temperature-dependent SWIR offset drift, and residual chromatic aberrations that affect spatial registration across bands. Additionally, conclusions on discrimination are conditioned by the limited spectral dimensionality of the system (twelve discrete bands) and by the use of controlled tank experiments with simplified backgrounds and illumination. While these conditions enable reproducible and interpretable measurements, they do not fully capture the radiative complexity of natural aquatic environments.

## 7. Conclusions

A compact polarimetric multispectral imager operating across VNIR and SWIR bands was developed and characterized. The dual-channel architecture, combined with motorized spectral and polarization selection, enables end-to-end acquisition of 48 wavelength–polarization measurements per cube. The full calibration workflow, including radiometric correction, polarimetric equalization, optical compensation, and Stokes-based reflectance retrieval, proved robust and repeatable under outdoor illumination.

Spectral unmixing experiments demonstrated that endmember recovery is feasible even with a twelve-band library, with VCA achieving the best accuracy–complexity trade-off among the tested algorithms. Although reduced spectral dimensionality limits the recovery of weak or dark materials, combining VCA with the spectral–polarimetric database enables reliable unmixing and spatial recovery of polymers under the investigated conditions.

The PMSI system demonstrates that multispectral polarimetric measurements can enhance polymer discrimination in dry scenarios and partially improve detectability in shallow underwater environments under the controlled conditions investigated here. Discrimination among chemically similar polymers remains challenging and may require increased spectral resolution, improved SWIR sensitivity, or complementary sensing modalities [37,38].

All data processing and analysis were performed using MATLAB (The MathWorks, Inc., Natick, MA, USA) with custom-developed scripts implementing the calibration, polarimetric processing, and unmixing workflows described in this paper. Calibration coefficients, acquisition metadata, and algorithm configuration files were version-controlled. To ensure reproducibility, the software tools and libraries used in this study are publicly available and cited in the References section; processed datasets and scripts can be made available by the authors upon reasonable request.

## Figures and Tables

**Table 1:** Spectral and polarimetric performance of the PMSI system for each selected band.

$\lambda_{0}$	〈λ〉	Δλ	PER	${\%}_{M}$
430	430.01	+0.01	1400	63.95
490	489.51	−0.49	2161	72.30
550	549.07	−0.93	2928	76.27
630	633.59	+3.59	4071	74.26
700	701.74	+1.74	5192	66.87
750	749.51	−0.49	4540	58.39
930	928.79	−1.21	2803	9.50
1000	1001.00	+1.00	3295	35.05
1200	1203.23	+3.23	4917	49.74
1300	1298.01	−1.98	5817	55.80
1570	1569.57	−0.43	7449	68.75
1650	1650.74	+0.74	8332	61.23

## Data Availability

The data presented in this study are available on request from the corresponding autor.
